# Phenotype-genotype association for the identification of resistance to stem borers (*Diatraea* spp., Lepidoptera: Crambidae) in sugarcane

**DOI:** 10.3389/fpls.2026.1895116

**Published:** 2026-09-22

**Authors:** Claudia Echeverri-Rubiano, Carolina Saavedra-Díaz, Monica Prias-Blanco, Jhon Henry Trujillo-Montenegro, Hugo Arley Jaimes, Carolina Camargo-Gil, John Jaime Riascos, Fernando S. Aguilar

**Affiliations:** 1 Entomology Area, Variety Program, Centro de Investigación de la Caña de Azúcar de Colombia, CENICAÑA, Cali, Colombia; 2 Biotechnology Area, Variety Program, Centro de Investigación de la Caña de Azúcar de Colombia, CENICAÑA, Cali, Colombia; 3 Variety Program, Centro de Investigación de la Caña de Azúcar de Colombia, CENICAÑA, Cali, Colombia

**Keywords:** Diatraea spp., genome-wide association study GWAS, marker validation, resistant genes, stem borer, sugarcane, variety resistance

## Abstract

Sugarcane (*Saccharum* spp.) is a major crop for sugar production, but its productivity is affected by stem borers (*Diatraea* spp.). These insects cause significant losses by damaging the stem and disrupting the transport of photosynthates and nutrients. Although biological control has been effective in Colombia, the identification of genetic resources for the resistance would provide complementary long-term crop protection. This study aimed to identify markers associated with the resistance to insects. For this purpose, a population of 348 genotypes was evaluated by infesting 30 stalks per genotype with four 9-day-old larvae. The proportion of bored internodes, insect survival, and the Damage Survival Index were recorded for each stem. Genotypic data was obtained using GBS, RADseq, and WGS. A total of six marker–model associations, which includes 4 unique markers, were significantly associated with DS Index, but only 2 were effectively validated. Candidate genes were searched within a 353.1 kb window surrounding the validated markers, revealing 48 genes involved in signaling pathways, receptor-mediated recognition, metabolic responses, direct defense mechanisms, and structural modifications. The DS Index variable integrates plant damage and insect performance, providing a robust metric to study resistance. These results provide a foundation for the identification of genetic resources for *Diatraea* spp., with key components including receptor-like kinases involved in herbivore recognition, calcium-dependent signaling cascades, and regulatory elements that finely balance immune activation through both positive and negative mechanisms. The validated SNPs, together with the functional annotation of nearby genes, represent an innovative approach to incorporate molecular tools into sugarcane breeding such as genomic selection, genome editing and/or genetic transformation.

## 1 Introduction

Sugarcane (*Saccharum* spp.) is a major agronomic and industrial crop used for the production of sugar, ethanol, and energy worldwide, representing a key component of economies in tropical and subtropical regions (Heller-Uszynska et al., 2011; de Andrade Dutra Filho et al., 2023). Globally, sugarcane accounts for nearly 80% of table sugar production, with Brazil contributing approximately 49% of the world’s output (Sharma et al., 2012; Islam et al., 2021; Marin et al., 2021; FAO, 2025). Modern sugarcane cultivars possess a highly polyploid and aneuploid genome derived from interspecific hybridization between *Saccharum officinarum* and *Saccharum spontaneum*, two ancestral species with contrasting chromosome numbers and agronomic traits (Gouy et al., 2015). *Saccharum officinarum* (2n = 80) contributes with most of the genomic background and is characterized by high sucrose accumulation but increased susceptibility to biotic stresses, whereas *S. spontaneum* (2n = 40 to 128) provides genetic diversity and resistance to multiple stresses (Sharma et al., 2018). As a result, modern hybrids are estimated to contain approximately 80% of their genome from *S. officinarum*, 10% from *S. spontaneum*, and 10% from recombinant chromosomes (Heller-Uszynska et al., 2011; Racedo et al., 2016). This genomic complexity poses significant challenges for genetic analysis and breeding, particularly for the identification and functional interpretation of candidate genes associated with complex traits such as resistance to insect pests.

The sugarcane stem borer *Diatraea saccharalis* (Lepidoptera: Crambidae), is one of the most important insect pests affecting sugarcane and other grass crops in the Americas (Sidhu et al., 2013; Solis and Metz, 2016; Vargas et al., 2018). In Colombia, four main species (*D. saccharalis*, *D. indigenella*, *D. busckella* y *D. tabernella*) primarily affect sugarcane (Vargas et al., 2018; Echeverri-Rubiano et al., 2023). These insects cause significant economic losses in both sugar and ethanol production in the Cauca river Valley, Colombia (Tarazona Parra, 2011; Vargas et al., 2018; Barreto Triana et al., 2022). The damage caused by *Diatraea* occurs primarily within the stem, affecting plants from early developmental stages to harvest (Gómez et al., 2009; Atencio et al., 2017). Larvae create galleries inside the stalk, disrupting the transport of photosynthates and nutrients, reducing sucrose content, and weakening the structural integrity of the plant, leading to lodging in some cases (Gómez et al., 2009; Atencio et al., 2017). Despite its economic importance, the molecular mechanisms underlying resistance to *Diatraea* spp. in sugarcane remain poorly understood. This limitation highlights the need to understand resistance not only at the phenotypic level but also at the gene function and regulatory networks levels.

Plant resistance to insect herbivores involves a wide range of morphological, biochemical, and molecular mechanisms that counteract or compensate for damage (War et al., 2012). These defense responses are ultimately governed by complex gene networks that control perception, signal transduction, and downstream metabolic and structural changes (Rajesh Lather et al., 2025). Plant defenses are commonly classified as constitutive or induced, although they often rely on similar compounds, proteins and structural changes (Gatehouse, 2002). Constitutive defenses include physical and chemical barriers present before insect attack, including tissue toughness, pubescence, glandular and non-glandular trichomes, and allelochemicals with antifeedant, toxic, or repellent effects like cyanogenic glycosides, digestive enzyme inhibitors, lectins, glucosinolates, alkaloids, and/or terpenoids (Smith, 2005; Watts et al., 2023; Rajesh Lather et al., 2025).

Antixenosis in sugarcane stem-borer resistance may be influenced by both constitutive varietal traits and the physiological condition of the plant. In *Eoreuma loftini*, the presence of dry leaves and plant stress, mainly to drought, increased oviposition and infestation (Meagher et al., 1996; Reay-Jones et al., 2007), suggesting an interaction between tolerance to abiotic stress and susceptibility to stem borers. Reay-Jones et al. (2007) also associated resistance with the presence of specific essential amino acids in sugarcane and rice, indicating that chemical traits may affect both host selection and insect development. In *Diatraea saccharalis*, varietal differences in oviposition have been reported, with females preferentially selecting upper, young leaves without pubescence, and vigorous plants (Fuchs and Harding, 1978; Sosa, 1988; Showler et al., 2012). Similarly, the *Saccharum robustum* accession IM76–228 showed reduced oviposition preference and lower larval survival, suggesting the simultaneous expression of antixenosis and antibiosis (Pimentel et al., 2017). However, despite evidence of differential oviposition among genotypes, a consistent association between antixenosis and reduced stem-borer populations under field conditions has not been demonstrated (Tucker, 1933; Kyle and Hensley, 1970).

Antibiosis resistance to stem borers is expressed through plant traits that reduce larval establishment, survival, development, or adult emergence after infestation (Smith, 2005). From a chemical point of view, antibiosis induced defenses are initiated through receptor-mediated recognition of damage-associated molecular patterns and herbivore-associated elicitors present in insect oral secretions (Sanches et al., 2017; Sabino et al., 2019; Rajesh Lather et al., 2025). These signaling pathways are primarily regulated by phytohormones such as jasmonic acid (JA), salicylic acid (SA), and ethylene (ET), where JA is mainly associated with responses to chewing insects, while SA and ET are more commonly linked to biotrophic pathogens and sap-feeding insects (Falco et al., 2001; Smith, 2005; Sanches et al., 2017; Franco et al., 2019; Watts et al., 2023; Rajesh Lather et al., 2025). These pathways drive extensive metabolic reprogramming, including the redistribution of primary metabolites such as sucrose and amino acids, which provide carbon and nitrogen for the synthesis of defensive secondary metabolites like phenols, alkaloids, and terpenes (Sabino et al., 2019; Watts et al., 2023; Rajesh Lather et al., 2025). In addition, plants deploy direct defense mechanisms, including the production of specialized proteins such as proteinase inhibitors, which impair insect digestion and pathogenesis-related proteins, whose enzymatic activities including chitinase, chitosanase, and RNase contribute to herbivore inhibition (Franco et al., 2019; Rajesh Lather et al., 2025). From the structural point of view, in sugarcane, these traits contribute to the resistance by limiting larval entry and establishment, including greater rind hardness or stalk rigidity (Mathes and Charpentier, 1963; Martin et al., 1975; Salgado et al., 2022), shorter periods of internode development (Martin and Cochran, 1975), tightly clasping leaf sheaths (Coburn & Hensley, 1972), and high fiber content (White et al., 2006). In parallel, structural modifications strengthen plant resistance through cell wall reinforcement, including lignification, suberization, and callose deposition, as well as the development of physical barriers such as spines, thorns, and dense trichomes that limit insect feeding, movement, and oviposition (Franco et al., 2019; Watts et al., 2023; Rajesh Lather et al., 2025). Together, these processes highlight that plant resistance is a coordinated outcome of signaling pathways, receptor-mediated recognition, metabolic responses, direct defense mechanisms, and structural modifications, all of which are tightly regulated at the gene level and contribute to the overall resistance or susceptibility.

Differences in resistance among sugarcane cultivars may be driven primarily by reduced neonate establishment rather than by differences in oviposition preference, with resistant cultivars showing lower injury under both greenhouse and field conditions and, in some cases, reduced larval growth and delayed pupation (Salgado et al., 2022). These findings indicate that antibiosis may be a major component of resistance to *D. saccharalis*, although some genotypes may simultaneously express antixenosis and antibiosis, thereby limiting both host acceptance and subsequent larval establishment and development (Pimentel et al., 2017). For instance, in the United States, NCo 310, CP 70-321, and HoCP 85–845 produced lower adult emergence of *D. saccharalis* and *E. loftini* (Bessin et al., 1990), whereas in South Africa, resistant cultivars N21, N29, and N33 reduced larval survival and adult production of *Eldana saccharina* compared with the susceptible cultivar NCo 376 (Keeping, 2006). In Brazil, IACSP94–2094 was particularly unfavorable for larval penetration and development (Dinardo-Miranda et al., 2012), while RB971747 and RB922579 showed lower infestation intensity and damage than SP79–1011 under field conditions, although these responses varied with the environment (Costa, 2007). In Colombia, field and greenhouse evaluations of sugarcane germplasm from Cenicaña’s breeding program against *Diatraea* spp. identified contrasting resistance responses and supported the selection of the susceptible cultivar CC 93–3826 and the moderately resistant cultivar CC 93–3895 as reference genotypes based on their contrasting levels of plant damage and insect survival (Echeverri-Rubiano et al., 2017; Echeverri-Rubiano et al., 2018; Echeverri-Rubiano et al., 2023).

The breeding program implemented at the Colombian Sugarcane Research Center (Cenicaña) have increasingly incorporated molecular tools to improve selection efficiency. Among these, genome-wide association studies (GWAS) have been widely used to identify molecular markers associated with complex traits by linking phenotypic variation with genome-wide molecular markers (Marees et al., 2018; Saavedra-Díaz et al., 2024). In sugarcane, GWAS has been successfully applied to identify loci associated with traits of agronomic and economic importance, including resistance to diseases such as sugarcane yellow leaf virus, brown rust, and orange rust, as well as traits related to biomass, sugar content, and yield components (Debibakas et al., 2014; Gouy et al., 2015; Racedo et al., 2016; Barreto et al., 2019; Yang et al., 2020; Saavedra-Díaz et al., 2024). Despite these advances, studies focusing on resistance to *Diatraea* spp. remain scarce, highlighting a gap in understanding the genomic variation with the functional mechanisms underlying the resistance to this pest. Beyond marker detection, understanding the functional roles of related genes is critical to elucidate the biological mechanisms underlying plant defense and to support the development of improved cultivars through molecular breeding strategies such as genomic selection, genome editing, and/or genetic transformation. In this context, the present study aimed (i) to associate phenotypic and genotypic variation in genotypes from the Colombian Sugarcane Research Center (CENICAÑA), (ii) to identify candidate genes, and (iii) to functionally interpret the role of the candidate genes in the resistance or susceptibility to *Diatraea* spp. Emphasis was placed on interpreting the candidate genes within key biological processes, including signaling pathways, receptor-mediated recognition, transcriptional regulation and hormonal crosstalk, metabolic and biochemical responses, direct defense mechanisms, and structural modifications, providing a framework to link genomic variation with plant defense responses, as they constitute the main pathways governing plant defense responses.

## 2 Materials and methods

### 2.1 Plant material

The plant material consisted of a subset of 348 genotypes selected from a core panel of 519 genotypes maintained at CENICAÑA (**Supplementary Table 1**). The 348 genotypes were divided into two populations: discovery and validation. The discovery population consisted of 220 genotypes representing the sugarcane diversity panel of CENICAÑA (Jaimes et al., 2024). Briefly, this panel was composed of 98 genotypes that represents the genetic diversity of CENICAÑA’s germplasm bank, 31 genotypes members of the genus *Saccharum officinarum* (15 genotypes), *Saccharum barberi* (7), *Saccharum sinense* (5), *Saccharum spontaneum* (1), and *Erianthus* spp. (3), 58 genotypes classified as susceptible or resistant to the most crop-limiting pests and diseases in Colombia, and 33 genotypes with high relevance for the breeding program of CENICAÑA (Jaimes et al., 2024) (**Supplementary Table 1**). In contrast, the second population, referred as the validation population, was composed of 128 sugarcane elite parents (EP) widely used at Cenicaña across the breeding schemes (**Supplementary Table 1**).

### 2.2 Experimental design and data collection

Both populations (discovery and validation populations) were evaluated for their resistance or susceptibility to the sugarcane borer *Diatraea saccharalis* (Lepidoptera: Crambidae). The experimental workflow comprised two sequential phases: the establishment and growth of the genotypes under field conditions, and the artificial infestation and resistance evaluation under greenhouse. For the field phase, the genotypes were planted in single-row plots at the Estación Experimental San Antonio (03° 21’ 7.3”N, -76° 17’ 59.67” W, and 1030 meters above sea level), Florida, Valle del Cauca, Colombia. Field management was under conventional agronomic practices supplemented with biological control agents, including *Trichogramma exiguum* (75 in ha^-1^), *Lydella minense* (50 flies ha^-1^), *Cotesia flavipes* (6 g ha^-1^), and *Bacillus thuringiensis* (2000 g ha^-1^) releases from the third to the seventh month after planting to maintain low populations of *Diatraea* spp. Additionally, when necessary, fields were treated with chlorantraniliprole (100 ml ha^-1^ in drench) at 45 days after planting. For the greenhouse, 30 nine-months old stalks were harvested per genotype by cutting them in the I + 8 internode (Kuijper, 1915). The stalks were then individually transferred to plastic containers where artificial infestation and resistance evaluation were conducted using the protocol proposed by Echeverri-Rubiano et al. (2023). Briefly, each stalk was infested with four larvae of *D. saccharalis* at the intersection of leaf sheaths and stalks and left until the insect reached the third instar (Echeverri-Rubiano et al., 2023). Twenty-one (21) days after infestation, each stalk was dissected to measure the proportion of bored internodes (BI) and the proportion of insect survival (SInd).

Because of greenhouse capacity limitations, the 348 genotypes were evaluated in 30 groups containing 14 genotypes plus the susceptible (CC 93-3826) and resistant checks (Echeverri-Rubiano et al., 2023). For the discovery population the resistant check was CC 93-3895 (Echeverri-Rubiano et al., 2023), while for the validation population, it was the cultivar 43-78 (*Erianthus* spp. wild specie) because of plant material availability and recent advancements in the research of *Diatraea* complex at CENICAÑA. Both populations were calibrated using the same susceptible check (i.e., CC 93–3826 coded as S58), which has consistently exhibited high damage to *Diatraea* spp. in greenhouse and field evaluations (Echeverri-Rubiano et al., 2023). Although CC 93-3895 (coded as S59) was initially selected as the resistant check (Echeverri-Rubiano et al., 2023), the *Erianthus* genotype 43-78 (coded as S3) was used in the validation population because it provided a stronger resistant reference relative to CC 93-3826, improving the biological contrast across the response variables without changing the interpretation of the index. The resistant check was not used as a criterion to determine whether a group was suitable or unsuitable, rather, it served only as a biological reference for contrasting resistance responses within each population. Each group was planted under a completely randomized design with 30 replications or stalks per genotype.

### 2.3 Phenotypic statistical analysis

Data from each population was analyzed following the model:


$$y_{ij}=\mu +E_{i}+G_{j(i)}+e_{ij}$$

where y corresponds to the response variable of genotype j in the group of evaluation i, μ the overall mean, $E_{i}$ the group of evaluation, $G_{j(i)}$ the random effect for the genotype j nested in the group of evaluation i, and e the random residuals. BI and SInd were analyzed assuming a binomial distribution with logit link function. Best linear unbiased predictors (BLUP) were obtained for each genotype for subsequent analysis because the traits showed high within-genotype variability, wide damage ranges, and a non-normal distribution, making the fixed-effect estimates more sensitive to noise and extreme observations (Piepho et al., 2008). Furthermore, the experimental design was only partially connected across evaluations through common checks (CC 93-3826, CC 93-3895, and 43-78), so BLUPs provided more robust genotype predictions by shrinking estimates according to their precision (Piepho et al., 2008). All analyses were done using SAS Enterprise Guide 9.2 (SAS Institute Inc., 2010). Finally, the damage survival index (DS Index) was estimated by the BLUPs produced for BI and SInd. The DS index was calculated as the proportion of bored internodes plus the proportion of insects survival divided by 2 ( $DS\;Index=\frac{BLUP_{BI}+BLUP_{SInd}}{2}$), with lower values indicating a greater host plant resistance (Echeverri-Rubiano and Camargo, 2025). This composite index (DS Index) conserved the biological rationale of the previously published Damage Survival Ratio (DSR) proposed by Echeverri-Rubiano et al. (2023), which jointly considers plant damage and insect survival. However, the DSR is based on the relative rankings of the BI and SInd components within each experiment, making the score assigned to a genotype dependent on the other genotypes evaluated in the same experiment and susceptible to changes when a modification in the genotypes is produced. In contrast, the DS Index is calculated from adjusted genotypic values rather than ranks, making it less dependent on the composition of a particular experimental group and more suitable for comparisons across assays. Considering that the resistance to the stem borers in sugarcane is independent of the insect species (Vargas et al., 2018; Echeverri-Rubiano et al., 2023), this experiment used *D. saccharalis* because of its shorter life cycle and its ability of mass rearing.

Broad-sense heritability for BI and SInd was estimated separately within each evaluation using binomial generalized linear mixed models with a logit link and genotype fitted as a random effect. Cullis-type heritability (Schmidt et al., 2019) was calculated on the logit scale as:


$$H_{\text{Cullis}}^{2}=1-\frac{{\bar{v}}_{\text{ΔBLUP}}}{2{\hat{\sigma }}_{g}^{2}}$$

where ${\hat{\sigma }}_{g}^{2}$ is the estimated genotypic variance and ${\bar{v}}_{\text{ΔBLUP}}$ is the mean prediction-error variance of all pairwise differences between genotypic BLUPs. Heritability of the composite DS Index was derived from a joint binomial mixed model fitted to BI and SInd within each evaluation. The model estimated the genotypic variances of BI and SInd and their genetic covariance using the glmmTMB package (Brooks et al., 2017). Because the DS Index was defined as the equally weighted combination of the adjusted genotypic values of BI and SInd, its genotypic variance was calculated as:


$${\hat{\sigma }}_{g,DS}^{2}=\frac{1}{4}({\hat{\sigma }}_{g,BI}^{2}+{\hat{\sigma }}_{g,SInd}^{2}+2{\hat{\sigma }}_{g,BI,SInd})$$

The prediction-error variance of the DS Index was obtained from the joint prediction-error covariance matrix of the BI and SInd genotypic effects using the same weights. Cullis-type heritability for the composite index was then calculated. The estimates were obtained separately for each evaluation because most genotypes were assessed in a single assay and only CC 93–3826 and CC 93–3895 connected all evaluations. Therefore, in a combined analysis, genotype and evaluation effects would be only weakly connected, and comparisons between genotypes from different evaluations would depend mainly on indirect information. Estimating heritability within evaluations ensured that the genetic variance and the precision of genotype comparisons referred to the same experimental conditions and genotype set. Evaluation-specific estimates were summarized across assays using the median, interquartile range, mean, standard deviation, and range.

### 2.4 DNA extraction and sequencing

Genomic DNA was extracted following the protocol proposed by Dellaporta et al. (1983). For the discovery population, sequencing was performed using genotyping-by-sequencing, GBS (Elshire et al., 2011), restriction-site associated DNA sequencing, RADSeq (Baird et al., 2008), and whole-genome sequencing (WGS), whereas the validation population was sequenced using WGS only. Sequencing quality was evaluated with FastQC (Andrews, 2010). For each individual, cleaned reads from each sequencing technology were aligned independently to the monoploid reference genome CC 01-1940 (Trujillo-Montenegro et al., 2021) with Bowtie 2.2.5 (Langmead and Salzberg, 2012), and unique alignments were selected using SAMtools v.1.10 (Li et al., 2009). The resulting per-technology alignments of each individual were then combined into a single BAM file per genotype. A single joint variant-calling step was subsequently performed on these merged alignments with NGSEP 4.0.2 (Tello et al., 2022) for genotyping, variant detection, and SNP calling, assuming a ploidy of 10, a MAF of 1%, a calling rate of 75%, and a filter for biallelic SNPs. Because all samples were processed through the same alignment and calling pipeline with identical parameters, no cross-platform reconciliation of independent call sets was required. This produced a consensus panel of 121,166 SNPs across the 348 genotypes.

### 2.5 Population structure, kinship, and GWAS analysis

Population structure was inferred using discriminant analysis of principal components (DAPC) implemented in the adegenet package (Jombart, 2008) in R (R Core Team, 2019). While the present study focuses on 348 genotypes, the population structure was determined within the context of the core panel of 519 genotypes from CENICAÑA, ensuring a robust estimation of genetic clusters. The optimal number of subpopulations was determined based on the Bayesian Information Criterion (BIC) (Schwarz, 1978). To visualize the relationships among the DAPC assignments, Euclidean distances were calculated between genotypes using their posterior membership-probability profiles. These distances were subjected to hierarchical clustering using the average-linkage method, and the resulting dendrogram was displayed in a circular layout. The kinship was estimated with the VanRaden method (VanRaden, 2008) implemented in the statistical package GWASpoly (Rosyara et al., 2016). Genome-wide association analyses were made using the GWASpoly package with the general, additive, and dominant for reference and alternative allele models (Rosyara et al., 2016). Genomic inflation was evaluated independently for each of the 12 genetic models using the genomic inflation factor ( λ). For each model, the p-values were transformed into chi-square statistics using the appropriate degrees of freedom. For the additive and dominant models were evaluated using one degree of freedom, while, for the general model, marker-specific degrees of freedom were defined according to the number of estimable dosage classes. The inflation factor was calculated as proposed by Devlin and Roeder (1999):


$$\text{λ}=\text{median}[\frac{\chi_{i}^{2}}{\text{median}(\chi_{df_{i}}^{2})}]$$

where $\chi_{i}^{2}$ is the observed chi-square statistic for marker i, and $df_{i}$ is its corresponding number of degrees of freedom. Q–Q plots were examined separately for each genetic model. SNPs significantly associated with DS Index were identified using the false discovery rate (FDR) test with a significant level of 5% (Sesia et al., 2021). The apparent contribution of each significant SNP was initially estimated in the complete discovery population using Maddala (1983) pseudo- $R^{2}$. This statistic was calculated from the likelihoods of a reduced ( $\log L_{reduced}$) and a full ( $\log L_{full}$) model including the SNP effect:


$$R_{\text{Maddala}}^{2}=1-\text{exp}[\frac{2(\text{log}L_{\text{full}}-\text{log}L_{\text{reduced}})}{n}]$$

Where n is the number of individuals included in the analysis. Because the same population was used for marker discovery and effect estimation, these values were interpreted as apparent pseudo- $R^{2}$ estimates and may be affected by the Beavis effect and winner’s curse. Therefore, the predictive contribution of each identified SNP was evaluated using 250 random 80:20 training–testing partitions. For each partition, reduced and full linear mixed models were fitted to the training set. Both models accounted for population structure and kinship, whereas the full model additionally included the SNP as a fixed effect. Predictions were then generated for the testing set, and predictive performance was assessed using the predictive coefficient of determination ( $R_{\text{pred}}^{2}$). The incremental contribution of the SNP was calculated as $\text{Δ}R_{\text{pred}}^{2}=R_{\text{full}}^{2}-R_{\text{reduced}}^{2}$. Positive folds were defined as the percentage of partitions in which inclusion of the SNP increased $R_{\text{pred}}^{2}$.

The SNPs identified in the GWAS analysis were validated with the validation population by assessing their association with the DS Index. For this, a linear mixed model was fitted for each SNP, including the overall mean, the SNP genotype, and the population structure covariates as a fixed effect. Additionally, genetic relatedness among individuals was modeled through a random polygenic effect with covariance structure proportional to the genomic kinship matrix K, accounting simultaneously for population structure and kinship (Yu et al., 2006). Each SNP was tested under the same genetic model in which it was detected in the GWAS analysis (i.e., general, additive, or dominant models). The significance of each SNP marker was evaluated using Wald tests implemented in the sommer (Covarrubias-Pazaran, 2016) and aod (Lesnoff and Lancelot, 2012) packages, which provide a χ² p-value for each fixed effect. To account for multiple testing, the Wald-test p-values obtained for all SNP–model combinations evaluated in the validation population were adjusted using the Benjamini–Hochberg false discovery rate procedure (Benjamini and Hochberg, 1995). Associations with an FDR-adjusted p-value below 0.05 were considered successfully validated. Linkage disequilibrium (LD) decay analysis was performed to define the genomic window for candidate gene identification around each validated marker. Pairwise LD between markers was estimated in the discovery population as the squared of the correlation coefficient (r^2^) using the Lddecay function of the sommer package (Covarrubias-Pazaran and Covarrubias-Pazaran, 2023). Subsequently, the r^2^ values were plotted against the physical distance between marker pairs, and a locally weighted scatterplot smoothing (LOESS) regression curve with a span of 0.1 was fitted for each chromosome (Cleveland and Devlin, 1988). The LD decay distance was defined as the physical distance at which the LOESS curve intercepts with the r^2^ threshold of 0.1 (Cleveland and Devlin, 1988; Abdurakhmonov et al., 2008; Vos et al., 2017). Chromosome-specific estimates are presented in **Supplementary Table 2** and **Supplementary Figure 1**. The mean LD-decay distance across the ten chromosomes was 353.1 kb (**Supplementary Table 2**) and was therefore used as the genome-wide window for candidate-gene searches around validated markers. Finally, candidate genes were searched and the function of each one was manually evaluated using Interpro classification (Paysan-Lafosse et al., 2022), the Database for Annotation, Visualization, and Integrated Discovery, DAVID (Huang et al., 2009; Sherman et al., 2022), and STRING network (https://string-db.org/), keeping only genes involved in the plant response to herbivory attacks. These genes were visualized using JBrowse 1.16.1 informatic tool (Buels et al., 2016) with default parameters.

### 2.6 Functional enrichment and pathway analysis

Biological roles and molecular functions of the identified candidate genes were assessed through functional enrichment analysis using Gene Ontology (GO) terms (Consortium, 2026) and Kyoto Encyclopedia of Genes and Genomes (KEGG) (Kanehisa et al., 2008) pathway annotations implemented in DAVID Bioinformatics (Sherman et al., 2022) Resources. Candidate genes were mapped to GO functional categories and KEGG pathways to identify overrepresented biological processes, molecular functions, cellular components, and metabolic or signaling pathways among the candidate genes. Enrichment analyses were conducted using ShinyGO 0.85.1 (Ge et al., 2020) and DAVID (Sherman et al., 2022). Statistical significance was determined using Fisher’s exact test and *p-values* were adjusted for multiple testing using the Benjamini-Hochberg False Discovery Rate (FDR) procedure (FDR < 0.05) (Hochberg and Benjamini, 1990). Additionally, candidate genes were mapped to the Kyoto Encyclopedia of Genes and Genomes (KEGG) database to identify enriched metabolic pathways, with a focus on secondary metabolite and lipid biosynthesis pathways.

## 3 Results

### 3.1 Phenotypic analysis

Substantial variation in resistance to the sugarcane borer, *Diatraea* spp., was observed in both evaluated populations (**Figures 1**, **2**). Damage Survival Index (DS Index) ranged from 0.09 to 0.54 in the discovery population and from 0.11 to 0.53 in the validation population (**Supplementary Table 3**). Based on the combined occurrence of low plant damage and low insect survival, 31 genotypes in the discovery population and 20 genotypes in the validation population were classified as resistant (**Table 1**). Among these 51 genotypes, S2, S160, S132, S103, S200, S219, SCC88, and SCC116 showed DS Index values lower than or equal that of the resistant reference genotype S3 (43-78) (**Figure 3**). The proportion of bored internodes ranged from 12.03% to 50.28% per stalk in the discovery population and from 14.79% to 50.67% in the validation population (**Figures 1**, **2**). Similarly, insect survival, considering larvae, pupae, and adults, ranged from 6.11% to 59.48% in the discovery population and from 7.72% to 56.13% in the validation population (**Figures 1**, **2**).

**Figure 1:**
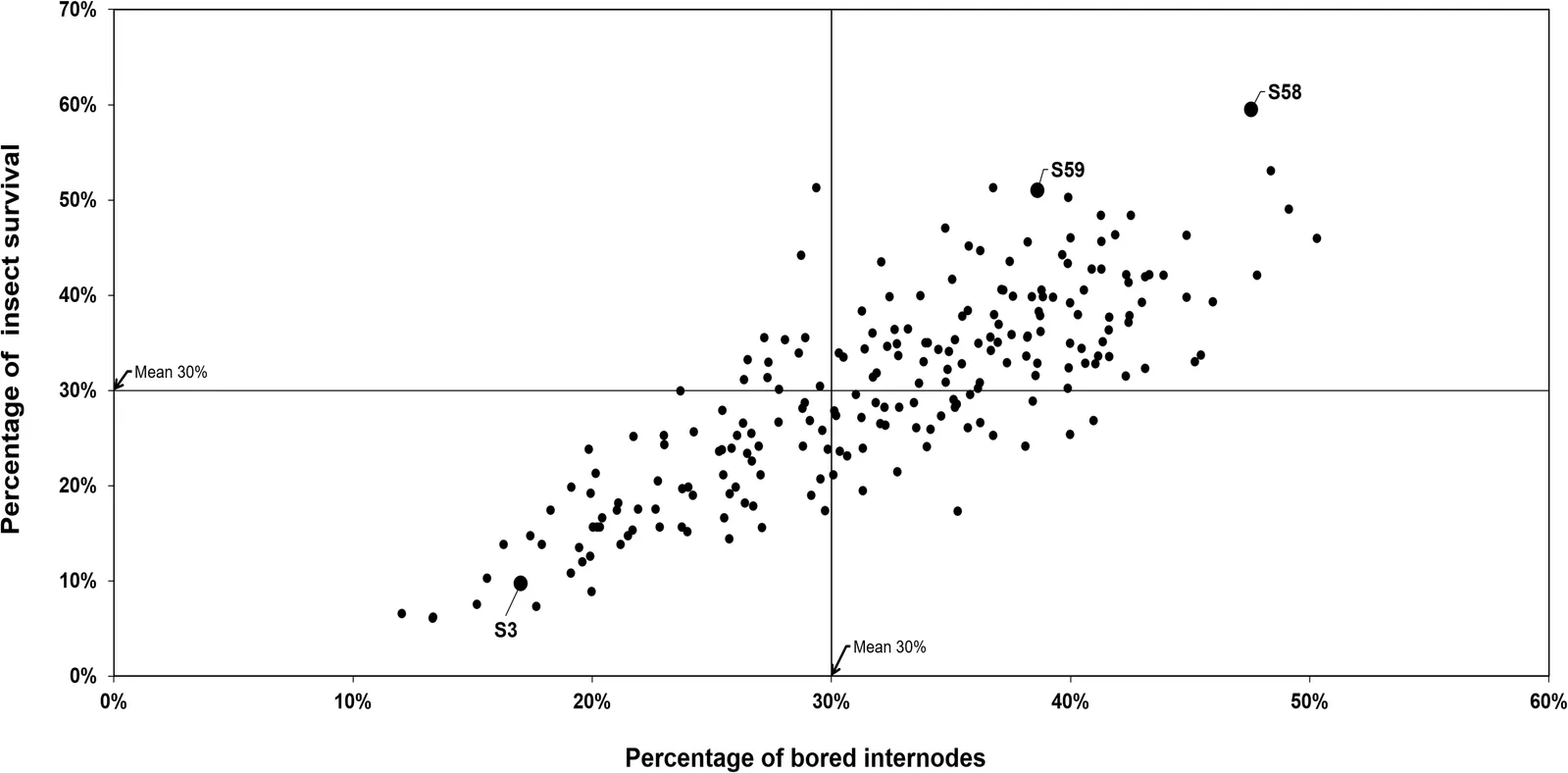
Mean percentage of bored internodes and mean percentage of insect survival (larvae, pupae and adults) for the discovery population. Data were analyzed as proportion with binomial, but they are indicated in percentage. The image highlights the susceptible S58 check, and the resistant checks S3 and S59.

**Figure 2:**
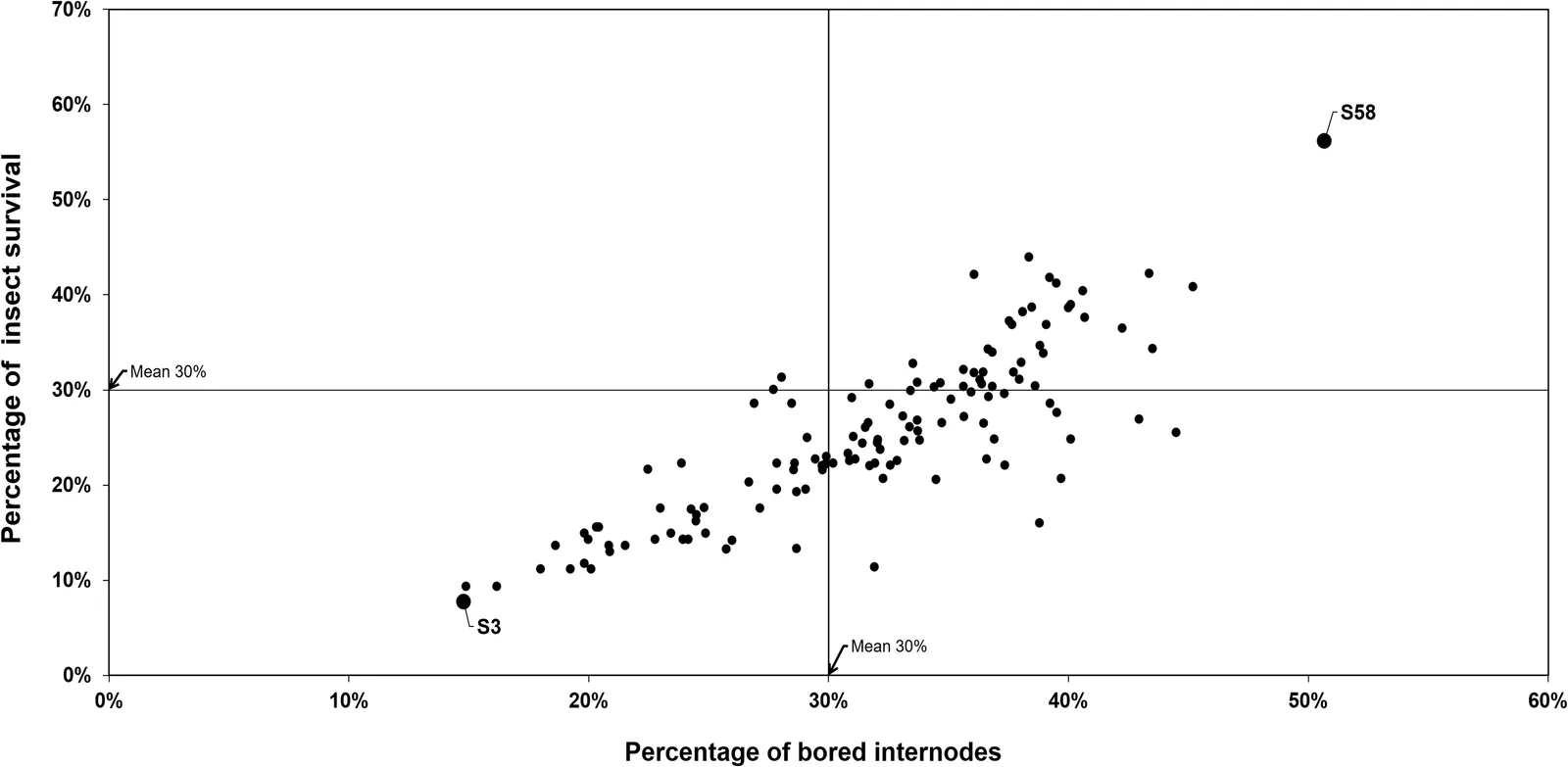
Mean percentage of bored internodes and mean percentage of insect survival (larvae, pupae and adults) for the validation population. Data were analyzed as proportion with binomial, but they are indicated in percentage. The image highlights the susceptible and resistant checks S58 and S3.

**Table 1:** Genotypes selected for their resistance to *D. saccharalis* when evaluated under controlled conditions.

Genotype	BI (%)	Sind (%)	DS index	Genotype	BI (%)	Sind (%)	DS index
S1	23.97	15.22	0.20	S200	12.03	6.58	0.09
S2	13.33	6.23	0.10	S204	18.24	17.43	0.18
S3	17.02	9.70	0.13	S210	20.41	16.63	0.19
S9	23.74	15.68	0.20	S216	19.09	10.86	0.15
S21	17.40	14.75	0.16	S219	15.59	10.29	0.13
S57	21.08	18.23	0.20	SCC3	24.86	14.97	0.20
S75	19.44	13.52	0.16	SCC35	20.82	13.70	0.17
S79	20.02	15.68	0.18	SCC54	17.97	11.22	0.15
S82	22.82	15.68	0.19	SCC71	19.21	11.20	0.15
S88	20.32	15.68	0.18	SCC72	20.30	15.65	0.18
S91	19.91	19.23	0.20	SCC77	22.75	14.34	0.19
S103	17.65	7.33	0.12	SCC84	18.61	13.71	0.16
S111	20.19	15.69	0.18	SCC88	16.16	9.43	0.13
S118	19.11	19.88	0.19	SCC93	19.96	14.35	0.17
S120	19.90	12.62	0.16	SCC99	19.79	15.00	0.17
S124	19.95	8.91	0.14	SCC116	14.86	9.43	0.12
S132	15.17	7.58	0.11	SCC117	20.41	15.65	0.18
S139	16.28	13.83	0.15	SCC129	23.41	14.98	0.19
S152	21.48	14.75	0.18	SCC136	19.80	11.83	0.16
S154	21.69	15.37	0.19	SCC142	25.72	13.33	0.20
S156	19.59	12.03	0.16	SCC145	21.51	13.70	0.18
S159	21.01	17.43	0.19	SCC154	23.91	14.33	0.19
S160	13.32	6.11	0.10	SCC156	20.07	11.22	0.16
S173	21.92	17.58	0.20	SCC166	20.86	13.06	0.17
S176	21.17	13.83	0.18	SCC182	24.14	14.33	0.19
S191	17.89	13.83	0.16				

These genotypes had a damage survival index (DS index) less than or equal to 0.20, a threshold associated with lower damage and reduced insect survival.

**Figure 3:**
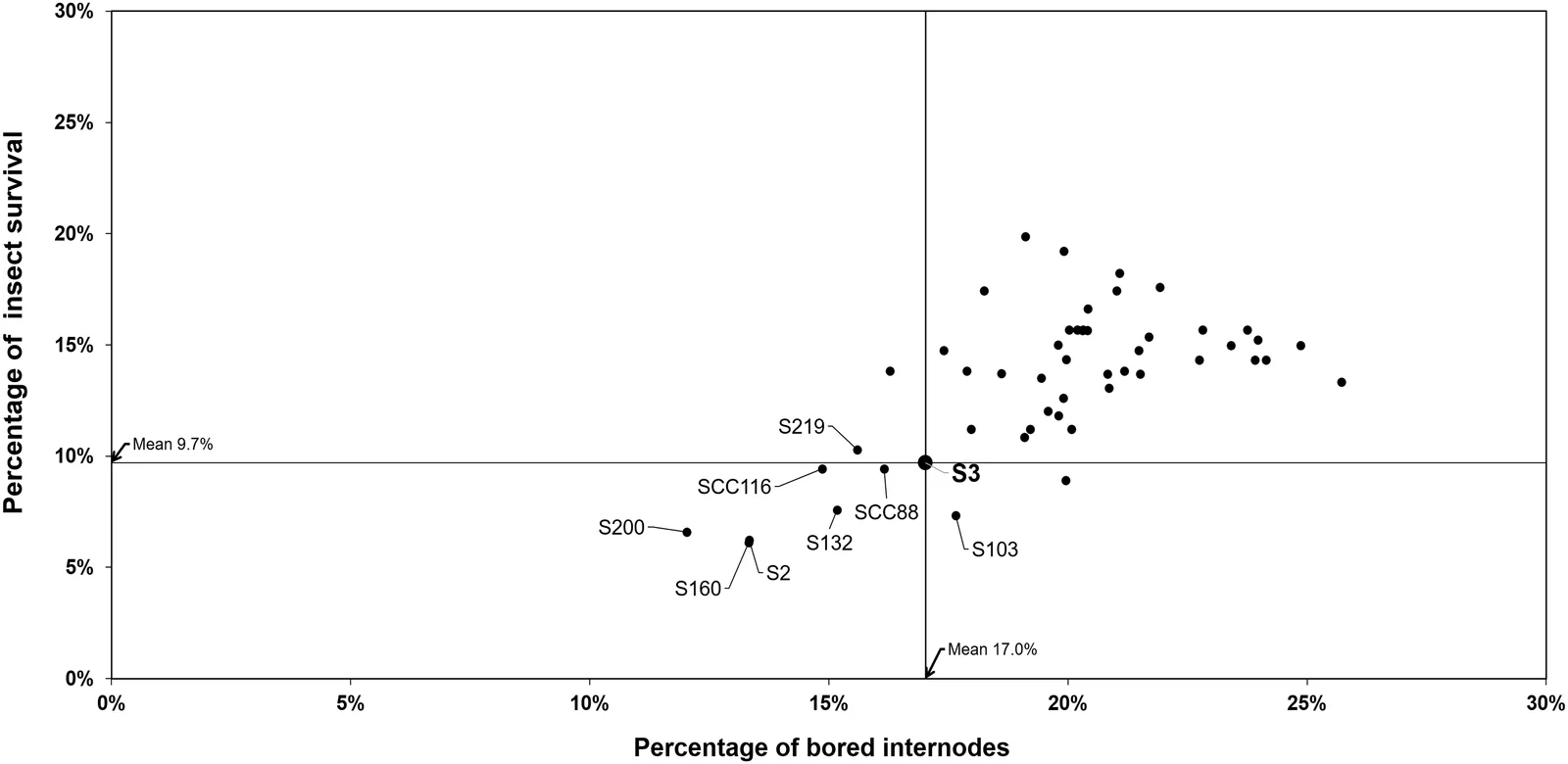
Resistant sugarcane genotypes identified based on the damage-survival index (DS Index) response to *Diatraea* spp. The image shows the 51 genotypes classified as resistant, with the selected genotypes highlighted for the use within the plant breeding program.

Broad sense heritability estimates differed among traits (**Table 2**). The DS Index showed the highest overall heritability, with a median of 0.89 and an interquartile range of 0.86–0.92 (**Table 2**). The mean heritability was 0.85 ± 0.16, with estimates ranging from 0.22 to 0.94 across evaluations (**Table 2**). SInd also showed relatively high heritability, with a median of 0.78, an interquartile range of 0.75–0.82, and a mean of 0.75 ± 0.15 (**Table 2**). In contrast, BI showed lower and more variable heritability across evaluations, with a median of 0.57, an interquartile range of 0.51–0.75, and a mean of 0.59 ± 0.19. BI estimates ranged from 0.14 to 0.84. Overall, the narrower interquartile ranges observed for the DS Index and SInd indicate greater consistency across evaluations than for BI. These results show that both BI and SInd contain genetically informative variation and support their integration into a biologically meaningful composite index. The high and relatively consistent heritability of the DS Index suggests that it captures the overall genotypic response to *Diatraea* with good precision, providing a suitable phenotypic basis for GWAS. Nevertheless, the limited population size and the likely polygenic architecture of the trait may still restrict the power to detect rare variants and loci with small effects.

**Table 2:** Broad sense heritability estimates for the composite damage survival index (DS index), proportion of bored internodes (BI), and proportion of insect survival (SInd).

Statistic	DS index	BI	SInd
Median	0.89	0.57	0.78
Q1	0.86	0.51	0.75
Q3	0.92	0.75	0.82
Mean	0.85	0.59	0.75
SD	0.16	0.19	0.15
Min	0.22	0.14	0.23
Max	0.94	0.84	0.95

Estimates were obtained separately within each evaluation and summarized using the median, first and third quartiles (Q1 and Q3), mean, standard deviation (SD), minimum, and maximum.

### 3.2 Population structure

The 519 sugarcane genotypes from the core panel at Cenicaña was structured into four subpopulations (**Figure 4**). The first subpopulation was predominantly composed of hybrid genotypes, with only a small representation of *Saccharum officinarum* and *Erianthus* spp. The second subpopulation mainly grouped ancestral and wild Saccharum species, including *S. officinarum*, *S. sinense*, *S. barberi* (with several subgroups), *S. spontaneum*, and *Erianthus* spp., with only a few hybrid genotypes (**Figure 4**). The third subpopulation was composed almost exclusively of hybrids, showing minimal contribution from ancestral *Saccharum* species (**Figure 4**). Finally, the fourth subpopulation was largely dominated by hybrids, although it also included several accessions of *S. officinarum*, *S. sinense*, and *S. barberi* (**Figure 4**). Some genotypes (SCC171, SCC325, SCC39, SCC279, and S52) exhibited similar posterior membership probabilities between subpopulations 1 and 3, which hindered an unambiguous assignment to a single group. These individuals showed intermediate ancestry coefficients, suggesting partial genetic contributions from both subpopulations. The 348 genotypes that were evaluated for the resistance/susceptibility to *Diatraea* spp. in this study were assigned to the four subpopulations described above (**Figure 4**), and the corresponding posterior membership probabilities were incorporated to control for spurious associations in the GWAS analysis.

**Figure 4:**
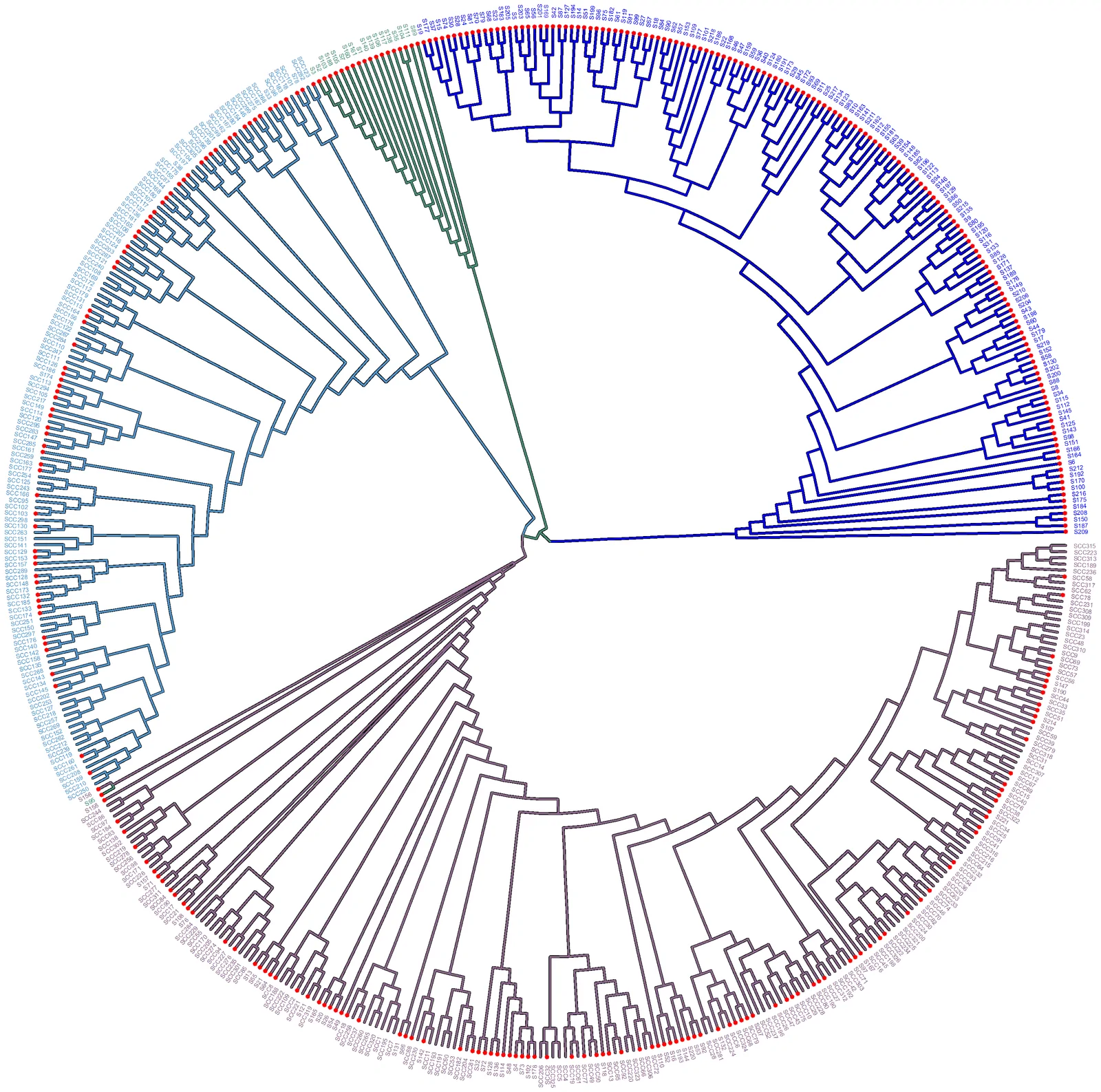
Structure analysis of 519 sugarcane genotypes from the core panel at Cenicaña, based on 121,166 SNPs. Subpopulations 1, 2, 3, and 4 are represented by pink, green, cyan, and blue colors respectively. Red dots above the genotype represent the subset of 348 samples used in this study.

### 3.3 Association analysis, marker validation and candidate genes

Genomic inflation factors were calculated separately for the 12 genetic models, with values ranging from 0.77 to 1.03 (**Table 3**). The highest value was observed for the general model ( λ=1.03), indicating only a minimal residual inflation, whereas some dominant models showed values below 1 (**Table 3**). Inflation factors close to 1 may reflect adequate control of population structure and relatedness, or subtle polygenic effects rather than substantial residual confounding (Yang et al., 2011). Because kinship and population structure were already accounted for in the mixed-model framework (Yu et al., 2006), no additional genomic-control correction was applied to the primary analysis. The Q–Q plot for the general model showed a mild but progressive upward deviation from the expected distribution, becoming more pronounced toward the upper quantiles (**Supplementary Figure 2**). Given the low magnitude of the corresponding inflation factor, this deviation may reflect the polygenic architecture of the trait, linkage disequilibrium between markers and associated loci, or the presence of multiple small-effect signals rather than severe residual confounding (Yang et al., 2011; Bulik-Sullivan et al., 2015). In contrast, λ values below 1 indicate and therefore applying genomic control to these models would increase the test statistics and could make the analysis artificially less conservative (Yang et al., 2011). As a sensitivity analysis, genomic control was applied to the general model using λ=1.03. After correction and recalculation of the FDR, no marker remained above the significance threshold, indicating that the associations detected under this model were close to the statistical threshold and sensitive to an additional conservative adjustment.

**Table 3:** Genomic inflation factor estimated for each of the 12 genetic models analyzed for DS index.

Modelo	Lambda
additive	1.01
general	1.03
1-dom-alt	0.98
1-dom-ref	0.77
2-dom-alt	1.00
2-dom-ref	0.89
3-dom-alt	1.00
3-dom-ref	0.91
4-dom-alt	1.00
4-dom-ref	0.96
5-dom-alt	1.00
5-dom-ref	0.99

Using the FDR-adjusted test, six significant marker–model associations were identified, corresponding to four unique SNPs (**Table 4**). SNPs 9:9208699_T/C and 10:7656049_T/C were each detected under two genetic models: the general (**Figure 5**) and 5-dom-alt (**Figure 6**) models for 9:9208699_T/C, and the general and 3-dom-alt (**Figure 7**) models for 10:7656049_T/C (**Table 4**). The remaining SNPs, 4:31216826_T/C and 3:18796998_T/A, were associated only with the general (**Figure 5**) and 5-dom-alt (**Figure 6**) models, respectively (**Table 4**). Apparent Maddala pseudo- $R^{2}$ values estimated in the full discovery population ranged from 11.27% to 20.82%, whereas the median cross-validated incremental predictive contribution $\left(\text{Δ}R_{\text{pred}}^{2}\right)$ ranged from 3.44% to 10.36% (**Table 4**). The highest median $\text{Δ}R_{\text{pred}}^{2}$was observed for 10:7656049_T/C under the 3-dom-alt model (10.36%), followed by 9:9208699_T/C under the 5-dom-alt model (10.13), while the lower median value was obtained for the 4:31216826_T/C (5.72%) and 9:9208699_T/C (3.44%) under the general model (**Table 4**). However, the interquartile ranges included negative values for all marker–model combinations, indicating variability in predictive contribution among partitions. Inclusion of the SNP improved prediction in 60.0% to 83.6% of the cross-validation folds (**Table 4**). Validation was conducted in an independent population using a linear mixed model that included population-structure covariates as fixed effects and a random polygenic effect based on the genomic kinship matrix. Of the six marker–model combinations evaluated, two were successfully validated: SNP 9:9208699_T/C and SNP 4:31216826_T/C both under the general model (**Table 4**). Overall, these results corresponded to two validated associations involving two of the four unique SNPs identified in the discovery population.

**Table 4:** Significant SNP–model associations for the Damage Survival Index (DS Index) in the discovery population.

Model	SNP ID	Marker	Apparent pseudo R^2^ (%)*	Cross-validated $\Delta R_{Pred}^{2}$, median (Q1;Q3)	Positive folds (%)	Wald-Test p-values	FDR P-value
general	CC-SNP4	10:7656049_T/C	13.91	9.85 (-12.4;27.41)	83.6	0.96	0.96
general	CC-SNP2	4:31216826_T/C	15.63	5.72 (-16.1;27.27)	71.6	6.40 x 10^-5^	3.84 x 10^-4^
general	CC-SNP3	9:9208699_T/C	20.82	3.44 (-24.8;28.5)	60	5.70 x 10^-4^	1.71 x 10^-3^
3-dom-alt	CC-SNP4	10:7656049_T/C	11.27	10.36 (-11.48;26.34)	82	0.63	0.76
5-dom-alt	CC-SNP1	3:18796998_T/A	11.48	9.34 (-13.67 to 25.03)	80	0.58	0.87
5-dom-alt	CC-SNP3	9:9208699_T/C	12.45	10.13 (-16.37 to 26.46)	82	0.26	0.52

The table includes the genetic model, SNP, apparent Maddala pseudo- $R^{2}$, cross-validated $\Delta R_{pred}^{2}$ as median [Q1–Q3], the percentage of folds in which the SNP improved prediction across 250 random 80:20 partitions, and Wald-test p-values from the independent validation population.

**Figure 5:**
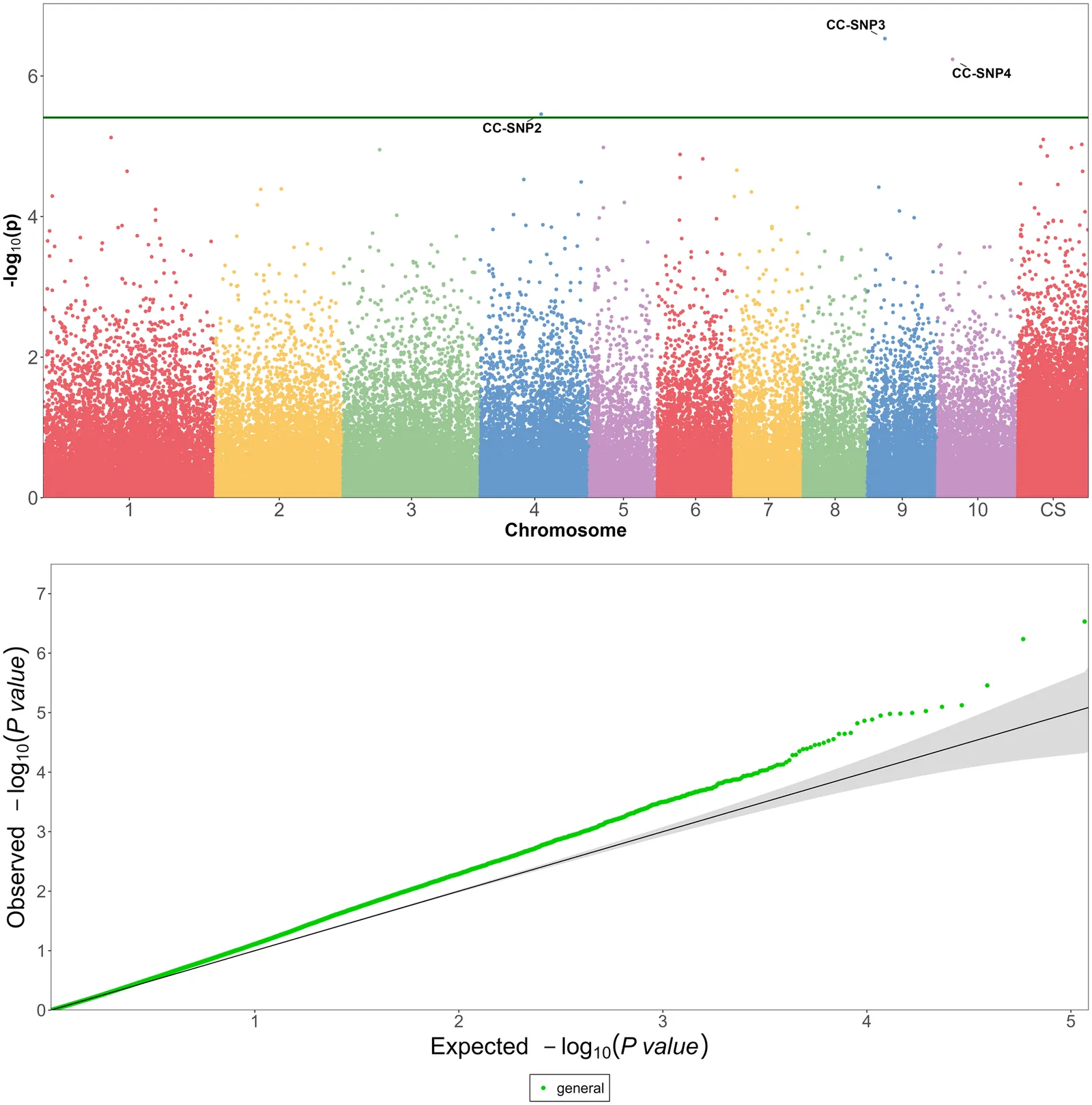
Manhattan and Quantile-Quantile plots for the damage survival index for the general model. The X-axis in the Manhattan plot corresponds to the chromosomes, with CS representing the collection of contigs and scaffolds, and the Y-axis shows values of -log_10_(p-value). The green line in the Manhattan plot represents the significance threshold.

**Figure 6:**
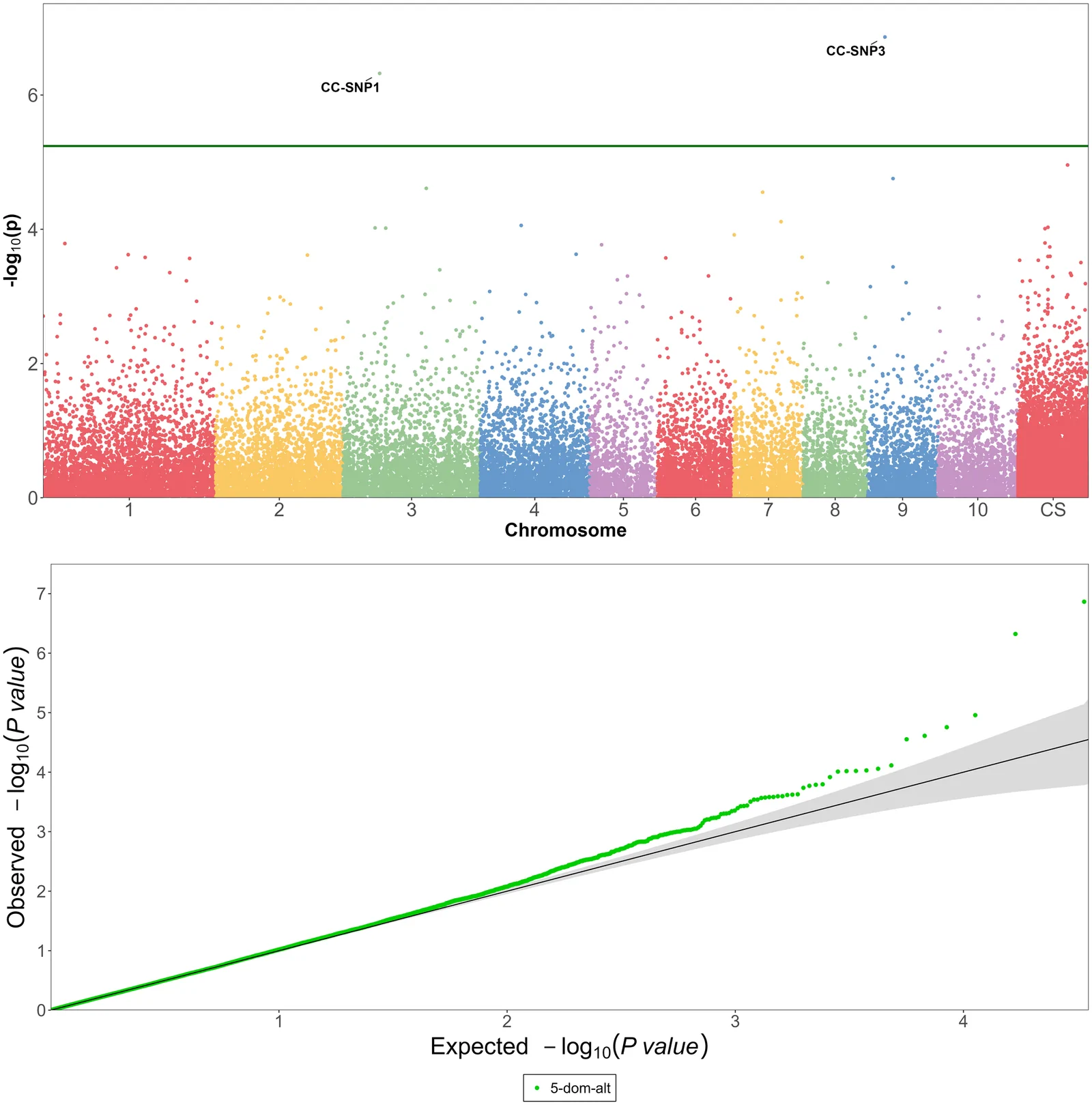
Manhattan and Quantile-Quantile plots for the damage survival index for the 5-dom-alt model. The X-axis in the Manhattan plot corresponds to the chromosomes, with CS representing the collection of contigs and scaffolds, and the Y-axis shows values of -log_10_(p-value). The green line in the Manhattan plot represents the significance threshold.

**Figure 7:**
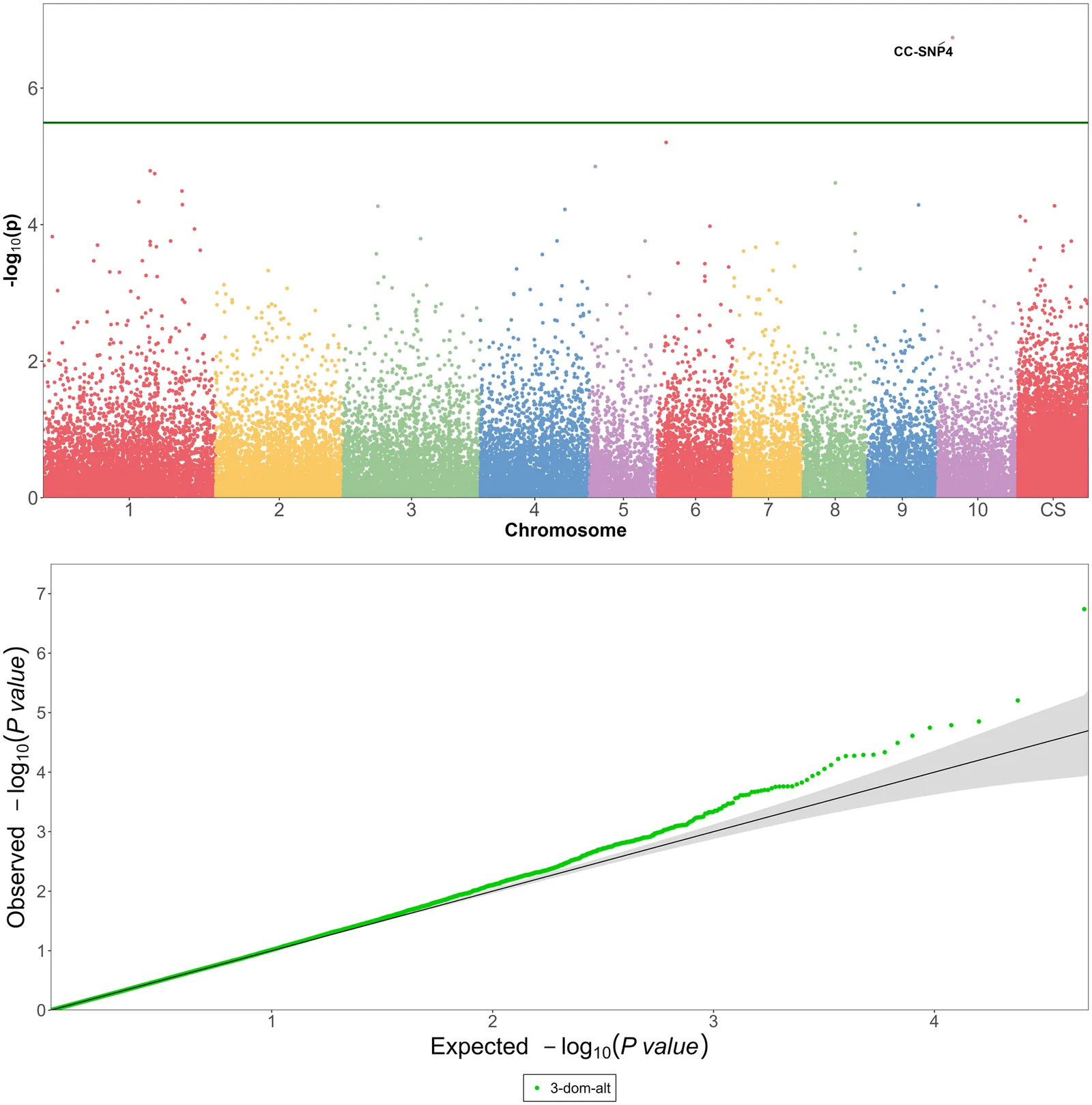
Manhattan and Quantile-Quantile plots for the damage survival index for the 3-dom-alt model. The X-axis in the Manhattan plot corresponds to the chromosomes, with CS representing the collection of contigs and scaffolds, and the Y-axis shows values of -log_10_(p-value). The green line in the Manhattan plot represents the significance threshold.

A total of 48 annotated genes were identified within the LD decay window of 353.1 Kb surrounding the two validated SNPs (**Supplementary Table 4**). These candidate genes were classified into five functional categories, including signaling pathways (27 genes), receptor-mediated recognition (3 genes), metabolic responses (12 genes), direct defense mechanisms (2 genes), and structural modifications (4 genes) (**Supplementary Table 4**). Given the large number of candidate genes, subsequent analyses focused on a subset of 11 selected genes (**Table 5**) based on the strength of their functional annotation, relevance to plant defense against herbivory, and consistency with previously reported mechanisms involved in responses to *Diatraea* attack (Huang et al., 2009; Franco et al., 2019; de Mello et al., 2020; Paysan-Lafosse et al., 2022; Sherman et al., 2022; Rajesh Lather et al., 2025). This prioritization allowed a more detailed and biologically meaningful interpretation of the genomic regions associated with DS Index.

**Table 5:** Candidate genes identified within a 353.1 kb LD decay-based window surrounding validated markers associated with resistance to *Diatraea spp*.

Functional categories	Gen	Gene name	SNP ID	Distance (Kbp)
Direct defense mechanisms	F3H	Naringenin,2-oxoglutarate 3-dioxygenase	CC-SNP3	-308
Metabolic responses	LPD	Dihydrolipoyl dehydrogenase%2C mitochondrial	CC-SNP3	-71
Metabolic responses	LPD1	Dihydrolipoyl dehydrogenase 1%2C chloroplastic	CC-SNP3	190
Receptor-mediated recognition	At3g58880	Putative F-box/LRR-repeat protein At3g58880	CC-SNP3	221
Receptor-mediated recognition	HERK1	Receptor-like protein kinase HERK 1	CC-SNP3	-198
Signaling pathways	ASR3	Trihelix transcription factor ASR3	CC-SNP3	92
Signaling pathways	PLIP3	Phospholipase A1 PLIP3%2C chloroplastic	CC-SNP3	-63
Signaling pathways	RS2Z32	Serine/arginine-rich splicing factor RS2Z32	CC-SNP3	-250
Metabolic responses	ESR1	Ethylene-responsive transcription factor ESR1	CC-SNP2	78.425
Signaling pathways	SD11	G-type lectin S-receptor-like serine/threonine-protein kinase SD1-1	CC-SNP2	-133.02
Metabolic responses	CYP75B3	Flavonoid 3’-monooxygenase CYP75B3	CC-SNP2	155.007

The distance column indicates the physical distance between each validated marker and the nearest candidate gene, with negative values indicating that the candidate genes are located downstream of the associated marker.

### 3.4 Functional enrichment analysis of candidate genes

The integrated functional enrichment analysis allowed the characterization of major biological, cellular, and metabolic processes associated with the evaluated candidate genes. According to the functional annotations (**Table 6**), the candidate genes could be grouped into four main functional categories. The first included the genes PMS1, SOG1, MBD13, ESR1, SEP1, and NRPE7, which were associated with DNA damage repair, DNA-binding activity, transcription, transcription regulation, and plant developmental processes (**Table 6**). The second group comprised the genes PYM, RS2Z32, and ECT2, which were annotated with RNA-binding, mRNA processing, mRNA splicing, spliceosome activity, mRNA transport, translation regulation, cell cycle, cell division, and mitosis, supporting the enrichment of pre-mRNA processing and spliceosome-related functions (**Table 6**). The third group was associated with cellular structure, cell wall remodeling, extracellular processes, and membrane-mediated signaling. This group included At1g48100 and CEP3, annotated as secreted hydrolases/proteases related to cell wall biogenesis/degradation and apoplast localization, as well as FLA2, HERK1, and NEK6, which were associated with membrane localization, receptor activity, kinase activity, and serine/threonine-protein kinase function (**Table 6**). Finally, the fourth group included the genes EMB1027, PDF1B, LPD1, F3H, and At1g09390, which were associated with chloroplasts, mitochondria, plastids, protein biosynthesis, lipid metabolism, flavonoid biosynthesis, hydrolase activity, ligase activity, and oxidoreductase activity (**Table 6**). Consistent with these annotations, KEGG pathway analysis revealed significant enrichment in primary energy and respiratory metabolism, including the citric acid cycle/TCA cycle (FDR: 1.5 × 10^-^²), pyruvate metabolism, glycolysis, and lipoic acid metabolism, supported by the oxidoreductase activity of organellar enzymes such as LPD1 and F3H (**Figure 8**).

**Table 6:** Functional annotation of candidate genes associated with resistance to *Diatraea spp*.

Gene	GO term category	Term categories
At1g09390	BP, CC, MF	Lipid degradation, Lipid metabolism, Secreted, Hydrolase
At1g48100	BP, CC, MF	Cell wall biogenesis/degradation, Cell wall, Secreted, Glycosidase, Hydrolase
At1g67900	BP	Ubl conjugation pathway
CEP3	CC, MF	Apoplast, Endoplasmic reticulum, Secreted, Developmental protein, Hormone, Hydrolase, Protease, Thiol protease
ECT2	CC, MF	Cytoplasm, Nucleus, RNA-binding
EMB1027	BP, CC, MF	Protein biosynthesis, Chloroplast, Mitochondrion, Plastid, Aminoacyl-tRNA synthetase, Ligase
ESR1	BP, CC, MF	Auxin signaling pathway, Cytokinin signaling pathway, Ethylene signaling pathway, Transcription, Transcription regulation, Nucleus, Activator, DNA-binding
F3H	BP, MF	Flavonoid biosynthesis, Dioxygenase, Oxidoreductase
FLA2	CC	Cell membrane, Membrane
HERK1	CC, MF	Cell membrane, Membrane, Kinase, Receptor, Serine/threonine-protein kinase, Transferase
LFR	BP, CC, MF	Growth regulation, Nucleus, Developmental protein
LPD1	CC, MF	Chloroplast, Mitochondrion, Plastid, Oxidoreductase
MBD13	BP, CC, MF	Transcription, Transcription regulation, Nucleus, DNA-binding
NEK6	MF	Kinase, Serine/threonine-protein kinase, Transferase
NRPE7	BP, CC	Transcription, DNA-directed RNA polymerase, Nucleus
PDF1B	BP, CC, MF	Protein biosynthesis, Chloroplast, Mitochondrion, Plastid, Hydrolase
PMS1	BP, CC	DNA repair and Nucleus
PYM	BP, CC, MF	Cell cycle, Cell division, DNA replication, Mitosis, mRNA transport, Nonsense-mediated mRNA decay, Plant defense, Translation regulation, Transport, Cytoplasm, Nucleus, Developmental protein, RNA-binding
RS2Z32	BP, CC, MF	mRNA processing, mRNA splicing, Nucleus, Spliceosome, RNA-binding
SEP1	BP, CC, MF	Differentiation, Flowering, Photosynthesis, Transcription, Transcription regulation, Chloroplast, Membrane, Nucleus, Photosystem II, Plastid, Thylakoid, Activator, Developmental protein, DNA-binding
SOG1	BP, CC, MF	Transcription, Transcription regulation, Nucleus, DNA-binding
UPL6	BP, MF	Ubl conjugation pathway, Ligase, Transferase

GO term categories correspond to biological process (BP), cellular component (CC), and molecular function (MF). Term categories list the main functional annotations assigned to each gene.

**Figure 8:**
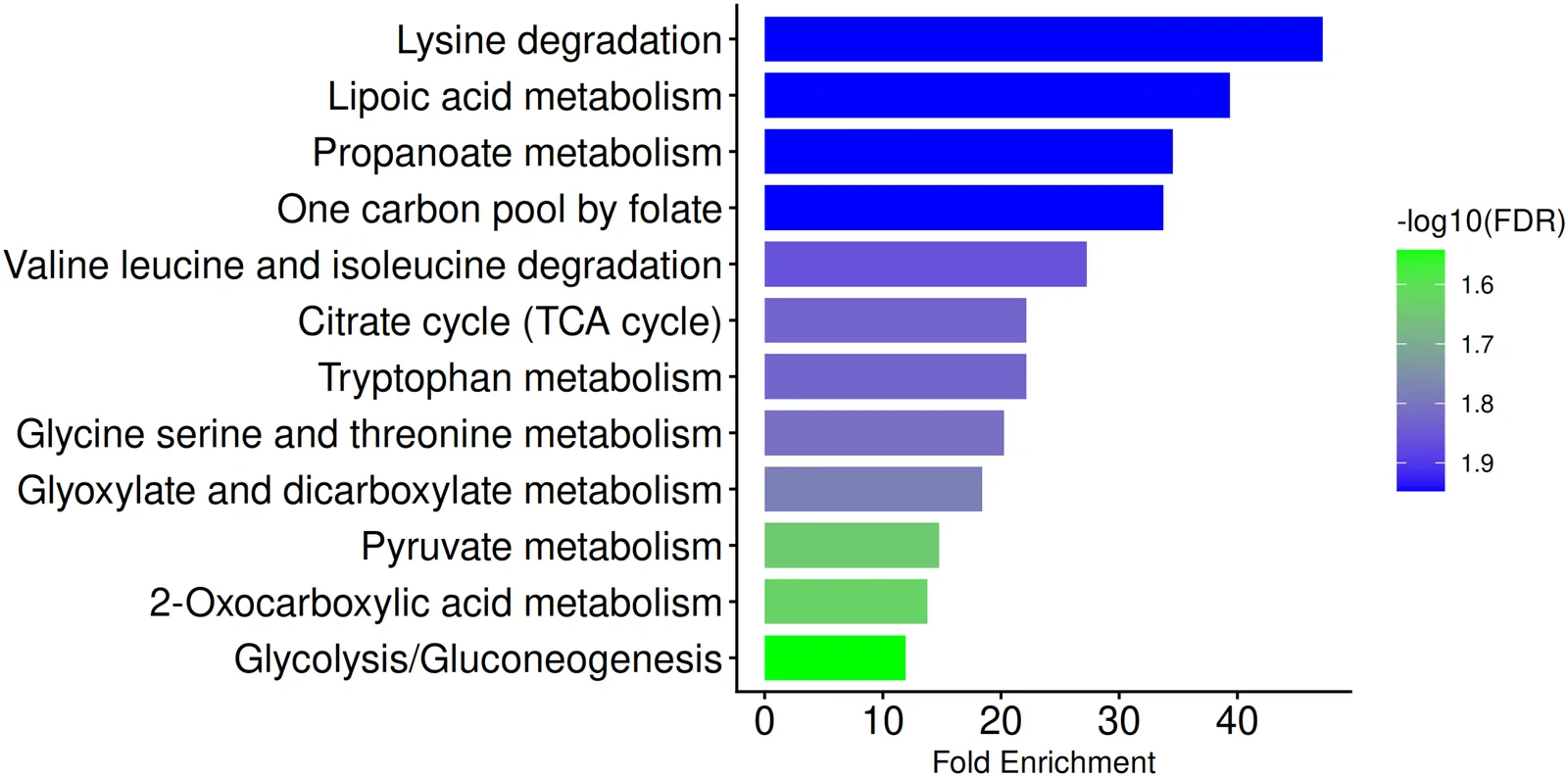
KEGG pathway enrichment analysis of candidate genes associated with resistance to *Diatraea* spp. Enriched pathways are shown according to their statistical significance, highlighting biological processes related to primary energy metabolism, respiratory metabolism, and defense-associated metabolic responses.

## 4 Discussion

### 4.1 Phenotypic analysis

Sugarcane borer, *D. saccharalis*, resistance is a complex trait that depends on the pest abundance as well as on several environmental conditions. These conditions could mediate the response of the plant to the insect and to differentiate the mechanisms the plant activate against the attack. For that reason, two main approaches have been developed to categorize resistance in sugarcane, one based on the damage to the plant and the other based on the survival of the insect (Bessin et al., 1990; Reay-Jones et al., 2003). Wilson et al. (2015) proposed the relative resistance ratio, which was applied for Salgado et al. (2022). This ratio is calculated by ranking the proportion of bored internodes and by ranking the relative survival as the proportion of the number of emergence holes over the number of internodes bored (Bessin et al., 1990). Therefore, they incorporate the damage to the plant (preference insect), and the survival of the insect assumed only its adult stage (prepupa, pupa, adult). Similarly, Echeverri-Rubiano et al. (2023) proposed the damage survival ratio (DSR) similar to Bessin et al. (1990) but including the insect in all its stages (larvae, prepupa, pupa and adult). The DSR is based on the relative rankings of these components within each experiment, consequently, the score assigned to a genotype depends on the other genotypes included in the same assay and may change when materials are added, removed, or replaced. For that reason. Echeverri-Rubiano and Camargo (2025) proposed the Damage Survival Index (DS Index) which is the modification of the DSR without using the ranking of the variables but rather making a proportion of bored internodes and survival stage. Therefore, the DS Index is calculated from adjusted genotypic values rather than ranks, making it less dependent on the composition of a particular experimental group and more suitable for comparisons across assays. All these indexes are applied mainly when there are resistant and susceptible cultivars along with large numbers of cultivars with high genetic diversity and with varying levels of resistance.

The implementation of the DS Index variable under greenhouse, where the infestation was completely controlled to guarantee pest pressure for insect-plant interaction and to prevent predation or parasitism by biological controllers, allowed the identification of 51 resistant genotypes with low damage and reduced insect survival (**Table 1**). Although check S59 (CC 93-3895) was initially selected as the resistant reference genotype for the discovery population, this designation was based on comparisons within a relatively limited set of evaluated genotypes in which it showed a clearly more resistant response than the susceptible reference S58 (Echeverri-Rubiano et al., 2023). However, as the number and diversity of evaluated genotypes increased, several accessions exhibited lower DS Index, BI and SInd values, than S59. Consequently, S59 was no longer sufficiently contrasting to serve as the resistant reference for the characterization of a diverse population. In contrast, the S3 genotype consistently showed a stronger and more clearly differentiated resistance phenotype, with lower plant damage and insect survival. For this reason, S3 was selected as the resistant reference genotype for further analysis at CENICAÑA. Eight (8) genotypes showed values of DS index below of the resistant genotype S3 (i.e., 43-78), indicating a high level of resistance to the sugarcane borer at CENICAÑA’s germplasm. These genotypes were representatives from the wild species *S. spontaneum* (S2), *S. barberi* (S160), *Erianthus* spp. (S132), *S. officinarum* (S103), and a hybrid selected from an early stage of selection at CENICAÑA (S200), and elite parents (SCC88, SCC116 and S219) (**Table 1**). These results showed a negative impact on the survival of *Diatraea*, and a low damage to the plant, suggesting a possible antibiosis-type resistance response in CENICAÑA’s germplasm (Smith and Clement, 2012). In the case of *S. spontaneum*, this specie, widely used within the breeding program of the USDA-ARS Sugarcane Research Unit, and has contributed in the breeding of promising sugarcane cultivars like Ho 08–9001 and Ho 08–9003 which have a known antibiosis type of resistance against the borer *Eoreuma loftini* (Dyar) (Lepidoptera: Crambidae) (White et al., 2011). The *S. barberi* species has been used as a parent in breeding program of ICAR-Sugarcane Breeding Institute in Coimbatore, India and USDA-ARS Sugarcane Research Unit because of its tolerance to abiotic stresses (Amalraj et al., 2011; Reed et al., 2012; Shrivastava and Srivastava, 2016) and perhaps has contributed indirectly to the tolerance or resistance to biotic stress such as that caused by *D. saccharalis*. Finally, S132 belongs to the *Erianthus arundinaceus* family, which has been characterized by its high biomass, thick vegetative stem, drought tolerance, and its resistance to several diseases and pests (Amalraj et al., 2011; Shrivastava and Srivastava, 2016). The pest resistance observed within the genus *Erianthus* can be explained by the production of the proteinase inhibitors (PIs) trypsin in the leaves, meristems, and stalks tissues, which highly affects the survival of *D. saccharalis* (Punithavalli, 2022; Punithavalli et al., 2024). For instance, clone 28NG7 (*E. arundinaceu*s) is used as a perimeter trap cropping to reduce the larvae survival and the damage of the borer *Chilo sacchariphagus* (Lepidoptera: Crambidae) in Asia (Nibouche et al., 2012, 2019). These results provide robust evidence of resistance within CENICAÑA’s germplasm and underscore the strategic importance of these eight genotypes as key parental lines for the development of varieties with enhanced resistance or tolerance to stem borers in Colombia.

### 4.2 Population structure

The core panel of 519 genotypes used in this study was structured into four subpopulations, of which subpopulations 1, 3, and 4 were primarily composed of varieties developed in Colombia (**Figure 3**). Within these groups, the genotypes S137, S94, S171, S144, and S216 stands out because of its use as progenitors in CENICAÑA. Among them, the S144 (POJ 2878) has been extensively used worldwide as a progenitor, driven by its high yield potential and broad adaptability to diverse environments (Orozco et al., 2015). The second subpopulation mainly grouped wild species, which is consistent with the evolutionary relationships within the *Saccharum* complex, as *S. spontaneum* has contributed approximately 39% of the genome of *S. sinense* and *S. barberi*, and between 15% and 27.5% of the genome of modern sugarcane cultivars (Piperidis et al., 2010; Cheavegatti-Gianotto et al., 2011; Orozco et al., 2015; Ali et al., 2019; Xiong et al., 2022). Similarly, the presence of *Erianthus* spp., one of the genera most closely related to *Saccharum*, highlights the genetic affinity among these taxa and their relevance for improving traits such as biomass production, vigor, ratooning ability, and tolerance to drought and waterlogging (Mukherjee, 1954; Wu et al., 2014; Evans and Joshi, 2020). On the other hand, the genotypes SCC171, SCC325, SCC39, SCC279, S52, and S157 showed similar probabilities of assignment between subpopulations 1 and 3, suggesting shared ancestry or admixture events, a pattern commonly reported in model-based population structure analyses (Pritchard et al., 2000). According to Pritchard et al. (2000), individuals with ancestry proportions distributed across multiple populations reflect historical or recent gene flow among genetic groups. In sugarcane, such introgression patterns are expected due to its complex domestication history and the extensive interspecific hybridization within the genus *Saccharum*.

### 4.3 Association analysis and candidate genes

Plant resistance to insect herbivory is a complex process involving constitutive and induced defenses, including damage perception, early signaling, transcriptional reprogramming, and downstream defensive responses (Dodds and Rathjen, 2010; Smith and Clement, 2012; Monaghan et al., 2014; Su et al., 2018; Rajesh Lather et al., 2025). Defense activation is initiated by the recognition of insect elicitors present in oral secretions and damage-associated signals generated during feeding (Medeiros et al., 2016; Franco et al., 2019; Rajesh Lather et al., 2025). These signals are perceived by extracellular, transmembrane, and intracellular immune receptors, activating phosphorylation-dependent signaling cascades that regulate downstream gene expression (Samayoa López, 2014; Couto and Zipfel, 2016; Watts et al., 2023; Rajesh Lather et al., 2025). In sugarcane, previous studies have shown that infestation by the sugarcane borer induces transcriptional responses within 12 to 48 hours, including up-regulation of jasmonic acid (JA), ethylene (ET), and defense-related genes, together with down-regulation of photosynthesis and energy metabolism pathways (Franco et al., 2019; de Mello et al., 2020). In this study, 48 were functionally associated with signaling pathways, receptor-mediated recognition, transcriptional regulation, hormonal crosstalk, metabolic responses, direct defense mechanisms, and structural modifications (**Supplementary Table 4**). From this group, 11 genes were selected and discussed in detail due to their biological relevance, while the remaining genes were not considered in the final interpretation. It is advised that these genes should be interpreted as positional and functional candidates rather than causal genes, since their contribution to DS Index was inferred from marker proximity and functional gene annotations.

#### 4.3.1 Signaling pathways and receptor-mediated recognition

During the initial recognition of attack, pattern recognition receptors (PRR) detect signals derived from damaged tissue and herbivore oral secretions, activating responses that share a common signaling basis (Furstenberg-Hagg et al., 2013; Zhou and Zhang, 2020; Snoeck et al., 2025). After the recognition, there is a rapid production of reactive oxygen species (ROS), along with the activation of complex intracellular signaling networks involving Ca²^+^ fluxes, MAPK cascades, and calcium-dependent protein kinases (CDPKs), all of which converge in transcriptional reprogramming, promoting the expression of defense-related genes (Tena et al., 2011; Macho and Zipfel, 2014; Su et al., 2018; Franco et al., 2019; Deshpande et al., 2025). These early signaling events are essential for the amplification and coordination of downstream defense responses.

Candidate genes encoding leucine-rich repeat (LRR)-containing proteins, including receptor-like proteins (RLPs), receptor-like kinases (RLKs), and F-box/LRR proteins such as *HERK1*, *At3g58880 and SD11* (**Supplementary Table 4**), were identified within the genomic regions of the validated SNPs, suggesting their involvement in receptor-mediated recognition and early signaling processes. These genes may contribute to extracellular perception and signal initiation during stem borer attack. In particular, *HERK1*, a member of the CrRLK1 receptor-like kinase family, has been associated with cell wall integrity sensing and ROS regulation (Cheung and Wu, 2011; Reymond, 2021; Xie et al., 2022). Since *Diatraea* larval affects the stem, genes involved in cell wall surveillance could participate in the perception of tissue disruption and activation of damage-associated signaling pathways. In addition, *At3g58880*, may contribute to the regulation of jasmonate- and ethylene-related signaling pathways through its role in the SCF ubiquitin ligase complex, thereby influencing the activation of plant immune responses (Kipreos and Pagano, 2000; Hu et al., 2024). In addition to LRR-containing domain structures, Lectin receptor-like kinases (LecRLKs) represent a crucial class of cell-surface pattern recognition receptors (PRRs) in plants, complementing LRR-containing structures (Kaur et al., 2023). Within this group, the candidate gene SD11 (a G-type lectin S-receptor-like serine/threonine-protein kinase) functions as a key sensor in plant innate immunity, highlighting the role of lectin-mediated recognition in pest and pathogen sensing (Baloyi et al., 2017). As membrane sensors, LecRLKs recognize specific molecular patterns, such as bacterial lipopolysaccharides (LPS) acting as MAMPs (Baloyi et al., 2017), and mediate defense against herbivorous insects, notably through the *BPH3* cluster (*LECRK1*, *LECRK2*, and *LECRK3*), which directly contributes to resistance against *Nilaparvata lugens* (Liu et al., 2015).

At the level of signal transduction, the gene RS2Z32 was associated with phosphorylation cascades and calcium (Ca²^+^) signaling pathways, which are fundamental components of plant immunity (Hu et al., 2025; Rajesh Lather et al., 2025). In this regard, the ATPase encoded by this gene may contribute to regulating defense signaling in sugarcane, given its documented overexpression in resistant plants during stem borer attacks (de Mello et al., 2020). However, it remains unclear whether this response is directly associated with insect herbivory or indirectly linked to secondary pathogens such as *Colletotrichum falcatum*. In addition to positive regulators of defense signaling, mechanisms of negative regulation are also essential for maintaining immune balance. In this case, the transcription factor ASR3 (Arabidopsis SH4-RELATED 3), a negative regulator of pattern-triggered immunity (PTI) was identified. Upon phosphorylation through the MPK4 pathway, ASR3 represses the expression of defense-related genes (Hofmann, 2015; Li et al., 2015; Wang et al., 2023). Its presence in the analysis suggests that susceptibility to *Diatraea* spp. may be associated with excessive negative regulation of immune responses, limiting the activation of defense mechanisms against herbivore attack. This type of negative regulation is essential to prevent energetically costly responses, but under certain conditions it may favor insect survival (Couto and Zipfel, 2016).

#### 4.3.2 Hormonal signaling and metabolic responses to herbivory

The activation of ethylene-responsive transcription factors plays a fundamental role in plant responses to herbivore attack by regulating the expression of key defense genes (Lorenzo et al., 2003). Within this group, the candidate gene *ESR1* (*Ethylene-responsive transcription factor ESR1*/*ENHANCER OF SHOOT REGENERATION 1*) acts as a key molecular switch rapidly induced by wounding during insect feeding (Iwase et al., 2017). *ESR1* coordinates a dual mode of epigenetic and transcriptional regulation by repressing the JAZ5 protein and directly activating the *ASA1* gene, thereby promoting auxin biosynthesis for tissue repair (Lee et al., 2024). Furthermore, ethylene signaling interacting with jasmonic acid stimulates the production of secondary metabolites such as flavonoids, which act as toxins or digestibility reducers to deter herbivory (Onkokesung et al., 2010; Falcone Ferreyra et al., 2012).

At the metabolic level, candidate genes associated with lipid metabolism appear as prominent targets within the GWAS loci linked to *Diatraea* spp. resistance. In this sense, the candidate gene GDSL (At1g09390) lipases release fatty acids from damaged membranes that are converted into oxylipins and JA, linking tissue disruption with systemic defense signaling (Chepyshko et al., 2012; Kim et al., 2014; Shen et al., 2022). Complementing this lipid-mediated response, the induction of LPD after the attack of *Diatraea* indicates broader metabolic reprogramming aimed at strengthening defense pathways. This gene encodes a lipoamide dehydrogenase, a key enzyme in mitochondrial and plastidial multienzyme complexes, and its activation may be linked to the increased demand for biosynthetic precursors (Lutziger and Oliver, 2000; 2001). Increased plastidial isoforms may enhance the supply of acetyl-CoA and NADH required for *de novo* fatty acid synthesis (Mooney et al., 2002), reinforcing the central role of lipid metabolism in defense activation. Finally, the candidate PLIP3 (Phospholipase A1 PLIP3), plays a key role in supplying lipid precursors for oxylipin synthesis by releasing α-linolenic acid in the chloroplast, which triggers OPDA (*12-oxo-phytodienoic acid*) accumulation and activates the jasmonic acid (JA) defense pathway (Wang et al., 2018). This mechanism mirrors its homolog PLIP1, a thylakoid phospholipase that hydrolyzes chloroplastic phosphatidylglycerol to supply the free fatty acid pool required for JA-mediated responses (Wang et al., 2017). In this context, the induction of PLIP3 in sugarcane during *Diatraea saccharalis* attack suggests that the plant recruits this plastid lipase as a primary metabolic node to channel chloroplast lipids into rapid oxylipin defense production (Wang et al., 2018), ultimately restricting larval development and performance in plant tissue.

#### 4.3.3 Direct defense and structural changes

Direct defenses include the synthesis of secondary metabolites and specialized proteins such as proteinase inhibitors (PIs), which interfere with digestive processes in the insect gut and reduce pest growth and survival (Rajesh Lather et al., 2025). Simultaneously, plants reinforce structural defenses through anatomical and biochemical modifications, including the development of trichomes and the fortification of cell walls via lignification and suberization, increasing tissue toughness and reducing digestibility (Rajesh Lather et al., 2025). As a case in point, the *F3H-2* (flavanone-3-hydroxylase) gene acts as a critical node in the flavonoid pathway, driving the accumulation of anthocyanins and proanthocyanidins (tannins) (Li et al., 2024). These compounds function as chemical deterrents, acting as antifeedants or toxins that reduce the survival and growth of insect larvae (Howe and Jander, 2008; Watts et al., 2023; Li et al., 2024; Rajesh Lather et al., 2025; Woolfolk et al., 2025). Furthermore, the Cytochrome P450 CYP75B3 enzyme, corresponding to Flavonoid 3’-monooxygenase (F3’H) serves as a central node in the B-ring hydroxylation of flavonoids, driving the synthesis of ortho-dihydroxyflavonoids such as quercetin and luteolin (Treutter, 2006) (Simmonds, 2001). In graminaceous plants, these hydroxylated phenolic compounds act as potent antifeedant and reduce larval digestibility by directly interfering with digestive enzymes in the insect gut (Treutter, 2006). Structure-activity relationship studies on lepidopteran pests of Poaceae demonstrate that specific structural modifications, such as the addition of hydroxyl (-OH) groups, alter the stimulation of deterrent neurons in the insect’s sensilla, thereby modulating feeding behavior between intake inhibition and stimulation (Simmonds, 2001).

These findings reinforce that resistance to *D. saccharalis* does not rely on a single gene or pathway, but rather on the coordinated interaction of perception mechanisms, signaling pathways, regulatory components, and defense effectors. The coexistence of positive and negative regulators suggests that tolerance or susceptibility may depend on the balance between activation and attenuation of plant immunity. In this context, the candidate genes identified in this study provide valuable targets for future functional validation and for the development of molecular breeding strategies aimed at improving resistance in sugarcane. From a breeding perspective, these findings provided a valuable framework for the identification of genomic regions and candidate genes associated with resistance to *Diatraea saccharalis*. The validated SNPs, together with the functional annotation of nearby genes, represent promising targets for the incorporation of molecular tools into sugarcane breeding programs. In particular, the integration of these markers into marker-assisted selection for population improvement and/or genomic selection schemes, which could improve the efficiency of selecting resistant sugarcane genotypes. Furthermore, the identification of resistance sources within both wild species and advanced hybrids highlights the potential of combining traditional breeding approaches with genomic information to accelerate the development of cultivars with enhanced resistance to stem borers in Cenicaña.

### 4.4 Functional enrichment analysis

The enrichment analyses using DAVID (Sherman et al., 2022) and ShinyGO (Ge et al., 2020) confirm that the identified candidate genes constitute a coordinated immune network rather than independent loci. Biological Process (BP) and Molecular Function (MF) analyses highlighted essential catalytic and lipid-metabolic activities, prominently featuring lipid degradation and lipid metabolism driven by the candidate gene At1g09390 (GDSL-like Lipase/Acylhydrolase), while Cellular Component (CC) and BP analyses demonstrate a strong enrichment in the nucleus and spliceosome, supported by key transcriptional and post-transcriptional regulators including ESR1, SEP1, MBD13, and SOG1 (Transcription/DNA-binding), NRPE7 (RNA polymerase), and RS2Z32 (mRNA splicing). Furthermore, the candidate gene PYM connect mRNA transport with plant defense responses, collectively orchestrating the genome-wide reprogramming required for stress adaptation (Moore et al., 2011).

The functional enrichment in organelle-associated components, specifically the chloroplast, mitochondria, and plastid underpin the heightened energetic and biosynthetic demands of plant defense (REFERENCE). Within these compartments, the candidate LPD1 (*lipoamide dehydrogenase 1*) acts as a pivotal oxidoreductase connecting primary carbon allocation with defense signaling (Lutziger and Oliver, 2001). Consistent with this organellar localization, KEGG pathway analysis revealed a significant over-representation of primary energy metabolism, particularly the TCA cycle, pyruvate metabolism, and lipoic acid metabolism (Bolton, 2009). These metabolic pathways, supported by enzymes such as LPD1, provide ATP that help in reducing power and carbon skeletons required for cellular repair, signal transduction, biosynthetic activity, and the production of defense-related metabolites, including oxylipin precursors (Lutziger and Oliver, 2001). In parallel, secondary metabolic processes were significantly represented, specifically Flavonoid biosynthesis driven by the candidate F3H (Simmonds, 2001; Treutter, 2006; Falcone Ferreyra et al., 2012). Taken together, these functional enrichment patterns demonstrate that sugarcane coordinates direct chemical defense mechanisms (e.g., flavonoid accumulation) with organellar bioenergetics, RNA processing, and lipid-derived signaling (At1g09390-mediated pathways) to restrict larval feeding and endure tissue damage caused by *Diatraea* spp. (Erb and Reymond, 2019).

Although this GWAS analysis identified genomic regions and candidate genes potentially associated with resistance to *Diatraea* spp., their functional involvement remains to be experimentally validated. In particular, comparing gene-expression levels before and after infestation in resistant and susceptible genotypes would help determine whether these genes are constitutively expressed or induced in response to insect attack. Such validation, using transcriptomic approaches or targeted RT-qPCR assays, would strengthen the biological interpretation of the associated loci and clarify their potential roles in antixenosis, antibiosis, or tolerance mechanisms. Therefore, these analyses represent an important next step for confirming the functional relevance of the candidate genes identified in this study.

## 5 Conclusion

This study integrates controlled phenotyping, genome-wide association mapping, and SNP validation to identify genomic regions associated with resistance to *Diatraea saccharalis* in sugarcane. Importantly, previous studies conducted at Cenicaña have shown that resistance to *D. saccharalis* can be extrapolated to other species present in Colombia, including *D. busckella*, *D. tabernella*, and *D. indigenella*. In this context, the implementation of a controlled infestation system, together with the use of the Damage Survival Index, enabled the reliable identification of resistant genotypes, which have been prioritized within the Cenicaña breeding scheme. The subsequent GWAS analysis identified 4 associated SNPs, of which 2 were validated in an independent population, supporting their robustness and reproducibility. Furthermore, the functional characterization of candidate genes revealed a coordinated network of defense mechanisms involving perception, signaling, transcriptional regulation, metabolic reprogramming, and direct defense responses.

In general, these results highlight the polygenic and integrated nature of the resistance to stem borers and provide a set of validated markers and biologically relevant candidate genes that can be incorporated into molecular breeding strategies, including genomic selection, genetic transformation, and genome editing. In this way, the integration of phenotypic, genomic, and functional information establishes a solid foundation for the application of genomic tools within the Cenicaña breeding program, ultimately contributing to the development of sugarcane cultivars with improved resistance and more sustainable pest management.

## Data Availability

The datasets generated and/or analyzed during the current study are available in the repository "https://github.com/fsilvaag/GWAS_Diatraea_Sugarcane"https://github.com/fsilvaag/GWAS-Sugarcane-Sucrose.git.

## Supplementary material

Supplementary Figure 1 Chromosome-specific linkage disequilibrium (LD) decay curves. Pairwise r² values are plotted against physical distance (kb) for each chromosome. The LOESS-fitted curve (black line) represents the LD decay pattern, the horizontal red line indicates the r² = 0.1 threshold, and the vertical green line marks the estimated LD decay distance for each chromosome.

Supplementary Figure 2 Quantile–quantile (QQ) plots for the twelve genetic models evaluated for resistance to *Diatraea spp*. Each panel compares the observed and expected −log_10_(p) values to assess the distribution of association signals and the adequacy of population structure and relatedness correction in each model.
